# Films Based on Thermoplastic Starch Blended with Pine Resin Derivatives for Food Packaging

**DOI:** 10.3390/foods10061171

**Published:** 2021-05-23

**Authors:** Cristina Pavon, Miguel Aldas, Juan López-Martínez, Joaquín Hernández-Fernández, Marina Patricia Arrieta

**Affiliations:** 1 Instituto de Tecnología de Materiales (ITM), Universitat Politècnica de València (UPV), 03801 Alcoy, Spain; miguel.aldas@epn.edu.ec (M.A.); jlopezm@mcm.upv.es (J.L.-M.); 2 Departamento de Ciencia de Alimentos y Biotecnología, Facultad de Ingeniería Química y Agroindustria, Escuela Politécnica Nacional, Quito 170517, Ecuador; 3 Research Group in Polymer Science, Engineering and Sustainability, Esenttia, Mamonal Industrial Zona, km. 8, Cartagena 130013, Colombia; hernandez548@hotmail.com; 4 Department of Natural and Exact Sciences, Universidad de la Costa, Calle 58 # 55–66, Barranquilla 080002, Colombia; 5 Departamento de Ingeniería Química Industrial y del Medio Ambiente, Escuela Técnica Superior de Ingenieros Industriales, Universidad Politécnica de Madrid (ETSII-UPM), Calle José Gutiérrez Abascal 2, 28006 Madrid, Spain; m.arrieta@upm.es; 6 Grupo de Investigación: Polímeros, Caracterización y Aplicaciones (POLCA), 28006 Madrid, Spain

**Keywords:** bioplastic, thermoplastic starch, pine resin, gum rosin, disintegration, packaging

## Abstract

Completely biobased and biodegradable thermoplastic starch (TPS) based materials with a tunable performance were prepared for food packaging applications. Five blends were prepared by blending TPS with 10 wt%. of different pine resins derivatives: gum rosin (GR), disproportionated gum rosin (RD), maleic anhydride-modified gum rosin (CM), pentaerythritol ester of gum rosin (LF), and glycerol ester of gum rosin (UG). The materials were characterized in terms of thermo-mechanical behavior, surface wettability, color performance, water absorption, X-ray diffraction pattern, and disintegration under composting conditions. It was determined that pine resin derivatives increase the hydrophobicity of TPS and also increase the elastic component of TPS which stiffen the TPS structure. The water uptake study revealed that GR and LF were able to decrease the water absorption of TPS, while the rest of the resins kept the water uptake ability. X-ray diffraction analyses revealed that GR, CM, and RD restrain the aging of TPS after 24 months of aging. Finally, all TPS-resin blends were disintegrated under composting conditions during the thermophilic incubation period (90 days). Because of the TPS-resin blend’s performance, the prepared materials are suitable for biodegradable rigid food packaging applications.

## 1. Introduction

Synthetic plastics production and applications have experienced exponential growth since the beginning of the polymer industry on a large scale back in 1940 and 1950 [1]. Despite the widespread use of plastics as materials are relatively new in history, after World War II [1], they have become essential for the world economy and modern life activities [2]. Synthetic plastics provide many benefits as membranes for water purification, food packaging to prevent spoilage, or lightweight transportation to reduce fuel consumption [3]. However, the current linear-economy model is unsustainable as the production and disposal mechanisms of polymers did not consider their end-of-life issues [2]. Thus, there is a need for the plastic industry to move towards a circular economy model, particularly in the case of short-term applications. 

Polymeric waste causes worldwide environmental pollution and huge economic and material value loss [2,3]. Furthermore, the majority of synthetic plastics are not biodegradable, which has increased the disposed of polymer waste amount in the past decades [4,5]. Moreover, the annual production of plastics keeps increasing, from 15 million MT in the sixties it has reached 359 million MT in 2018 [6] and it is expected to triple by 2050 (reaching 1.12 billion tons) [2,5]. In this frame, many researchers and companies intend to produce sustainable polymers to gradually replace fossil-based polymers [2,5]. As a result, several sustainable polymers have been developed from renewable raw materials, for instance, biopolymers such as cellulose [7], lignin [8], plant-based fatty acids, or polymers from animal origin such as chitin [9], caseinates [10], etc. Among them, carbohydrates are convenient raw biopolymers due to their availability, inexpensiveness, and stereochemical diversity [11]. Moreover, defined properties of these biopolymers can be attained through their chemical modification and/or physical blending [12,13].

Starch is produced by plants such as wheat, corn, rice, bean, tapioca, and peas [14]. Starch is composed of two polysaccharides (amylose and amylopectin). Starch is widely used thanks to its low cost and high availability [15,16]. To use starch in the plastic industry, it can be plasticized in the presence of plasticizers, high temperatures, and shear stress [15]. Thermoplastic starch (TPS) has gained considerable attention during the last years for the development of biodegradable starch-based food packaging materials or edible coatings [17]. TPS is made from edible starch plasticized with food-grade plasticizers and thus allowed for food contact applications. Moreover, TPS is biobased and biodegradable, which from an environmental perspective allows closing the loop of circular economy [18]. However, TPS industrial application is somewhat limited due to its poor mechanical performance, low water resistance, and the undesirable changes in the thermomechanical characteristics of the material caused by the re-crystallization and retrogradation that its structure is subject to [18,19,20]. Thus, in addition to increasing its water resistance, improvements on the thermo-mechanical performance of TPS-based formulations are needed to extend its industrial applications [21]. To enable TPS industrial applications in the food packaging sector several strategies have been proven, such as chemical modifications [17], blending [19,22], and/or the development of composites and nanocomposites [23]. Melt-blending strategies seem to be an effective way to tailor TPS properties by a simple, industrially scalable, and cost-effective plastic processing method [18]. However, TPS-based formulations usually show the glass transition temperature above or below room temperature as a function of the plasticizer and the water content [23]. Above T_g_, the polymeric matrix loses its rigidity leading to plastic deformation making the material unsuitable for warm or hot food applications [18].

The revalorization of food and agro-industrial wastes into environmentally friendly materials has considerably increased during the last years [14,24]. In this sense, the revalorization of pine resin and gum rosin derivatives has gained interest in the food packaging field during the last years as natural low-cost additives (i.e., stabilizers, compatibilizers, and/or plasticizers) [18,19,25,26,27]. Resin is exudated from Conifers as a defense mechanism in wounds of their tissues or cuts of the wood of the stem [28]. During the last years, tapping pine trees to collect secretions of resin has resurgence [29,30], and among these, the activities related to this field, as pine cleaning activities during the summer period when fire risk is high [29]. Therefore, the revalorization of pine resin and gums derivatives are positive for good forest management practices. Concerning the plastic packaging industry, gum rosin is a natural and easily available material that has great potential in the development of blends with biopolymers. Gum rosin is a rigid and brittle solid that has a thermoplastic behavior [31]. Gum rosin is the non-volatile fraction of pine resin [32] and is composed mainly of abietic- and pimaric-type rosin acids that have characteristic hydrophenanthrene structures [33]. Rosin acids structure have conjugated double bonds and a carboxylic group which enable gum rosin the possibility to be chemically modified and be converted into a large number of derivatives such as salts, hydrogenated, esters, maleic anhydride adducts, and disproportionated rosins [33,34]. These modified rosins have different properties and are useful in several applications [33].

Gum rosin and its derivatives have been successfully used in the production of green plastics [34]. For instance, Arrieta et al. (2017) studied mixtures of triethylene glycol ester of gum rosin (TEGR) with linseed oil as natural additives in polyvinyl chloride (PVC). They have found that TEGR enhanced the tensile strength and the elastic modulus of the plastisol, and also, it provides a UV-blocking effect and contributes to increasing the thermal stability of PVC [29]. In 2019, Aldas et al. studied the effect of gum rosin and two derivatives (pentaerythritol rosin esters: LF and UT) on the performance of a commercial thermoplastic starch (Mater-Bi). They determined that gum rosin in 15 wt.% increases the elongation at break, toughness, and impact energy. Besides, LF in 10 wt.% increases the toughness, Young’s modulus, and tensile strength of Mater-Bi. Meanwhile, UT in 15 wt.% increases the elongation at break of neat Mater-Bi and improves its processability performance by decreasing the processing temperature [19]. Then, the same authors deepen in a microscopic study of the mentioned materials and found the interactions of gum rosin and the two pentaerythritol rosin esters over the different phases of Mater-Bi [35]. In another area, Pavón et al. (2020) studied the effect of gum rosin on poly(ε-caprolactone) (PCL) processed by 3D-printing, it was determined that GR form homogeneous blends with PCL, plasticized the structure, and increase the hydrophobicity of the material [36]. Thus, gum rosin and its derivatives have acquired considerable interest as a sustainable additive, and the formulated materials which contain them can be used in diverse applications such as food packaging, mulch films, biodegradable and compostable films, and biocompatible materials.

Starch has proven to be an interesting food-grade polymeric system able to develop environmentally friendly packaging products that contribute to reducing petrochemical plastic consumption as well as waste generation, being widely blended with industrial wastes [14]. To extend starch applications in the food packaging field, it can be prepared in the thermoplastic form which requires water that acts as a destructuring agent and its further gelatinization. Then, the high water content joined with the heat applied during processing produces the starch granule swelling and the starch gelatinization through the disruption of the granule organization [37]. In previous work, TPS was prepared through melt extrusion from food-grade corn starch, glycerol, and water. Glycerol has been selected as a plasticizer not only because it is food-grade and contributes to the reduction in biopolymers intrinsic brittleness, but also it is a by-product of biodiesel production being important from an environmental point of view to revalorize industrial sub-products into useful plasticizer [10,37]. Then, TPS was blended, by melt extrusion, with five pine resin derivatives: gum rosin (GR), maleic anhydride-modified gum rosin (CM), pentaerythritol ester of gum rosin (LF), disproportionated gum rosin (RD), and glycerol ester of gum rosin (UG). Specimens of the materials were obtained from the injection molding process simulating the industrial conditions. The used pine resin derivatives showed their ability to stiffen TPS structure and thus improve the mechanical performance of the specimens, increase the thermal stability, and shift the TPS glass transition temperature to higher values, leading to materials able to be used for rigid packaging intended for hot food applications [18]. 

In the present work, to address the interest of these materials for the biodegradable food packaging industry, the injected molded TPS-resin materials were characterized focusing on this field of application. Thus, the thermo-mechanical behavior, surface wettability, and color parameters were evaluated. The changes in the crystallinity due to the aging process were evaluated by X-ray diffraction pattern after 24 months of aging. Finally, the materials were disintegrated under composting conditions test was conducted in all TPS-resin blends to show that the materials close the loop and fit well the concept of materials for the circular economy approach. 

## 2. Materials and Methods

### 2.1. Materials

Native corn starch (food-grade) composed of 27% amylose was provided by Cargill (Barcelona, Spain). 

The plasticizers used were distilled water and glycerol, added in 10 wt.% and 25 wt.% respectively. Glycerol (99% of purity), was purchased from Panreac (Barcelona, Spain). 

**Figure 1:**
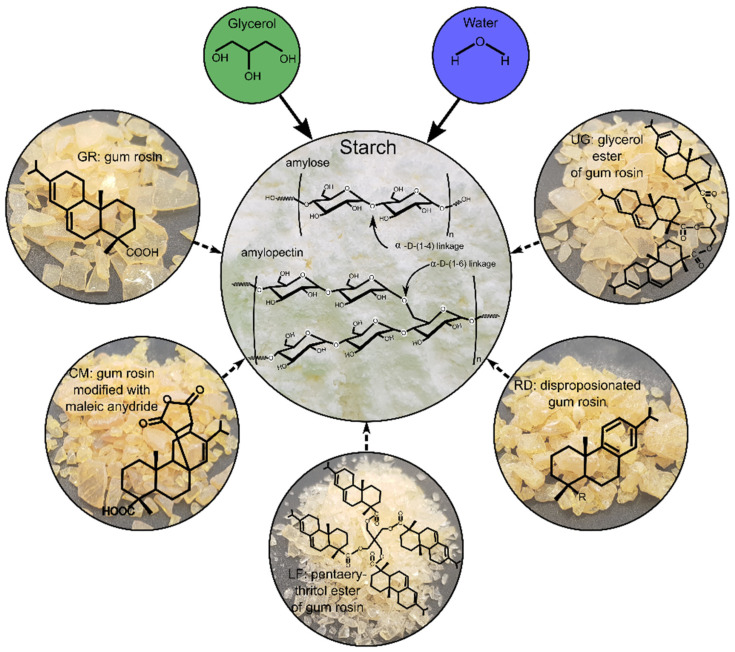
Chemical structure of gum rosin and gum rosin derivatives.

Gum rosin and four gum rosin derivatives were used as additives and added in 10 wt.% in the thermoplastic starch formulation: Gum Rosin (GR, softening point of 76 °C and acid number 167), supplied by Sigma-Aldrich (Mostoles, Spain); Colmodif R-330 (CM, softening point of 123 °C and acid number 252); Lurefor 125 resin (LF, softening point of 125 °C and acid number 11.9); Residis 455 (RD, softening point of 74.6 °C and acid number 157) supplied by Luresa (Segovia, Spain) and Unik Gum G88 (UG, softening point of 87 °C and acid number 7) supplied by United resins (Figueira da Foz, Portugal). Figure 1 shows the corresponding chemical formula of the used materials.

### 2.2. Methods

#### 2.2.1. TPS-Resin Blends

**Figure 2:**
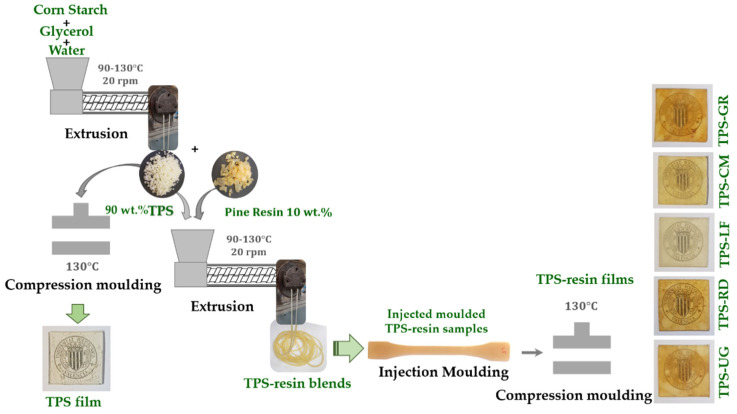
Schematic representation of TPS preparation and processing of TPS blends with gum rosin and gum rosin derivatives into injection molded and film samples.

TPS-resin blends were prepared by melt extrusion and the specimen samples were manufactured using injection molding, following the method already optimized in previous work [18]. A schematic representation of the thermoplastic starch (TPS) preparation starting from native corn starch, glycerol, and water, and further the development of TPS blended with gum rosin and its derivatives materials are shown in Figure 2. In brief, corn starch was manually mixed with water and glycerol, the resulting blend was hermetically stored in a sealed polyethylene bag for 24 h. To obtain TPS, the mixture was extruded in a co-rotating twin-screw extruder, at a temperature profile of 130, 110, 100, 90 °C (from die to hopper) and 20 rpm. The used extruder was from Dupra S.L (Castalla, Spain) equipped with a screw diameter of 25 mm and an L/D ratio of 24. After the extrusion process, each pine resin derivative was manually mixed with the corresponding amount of TPS, and the mixed materials were processed by a second extrusion. Neat TPS was also melt-extruded a second time to be used as a reference. The extruded materials were then pelletized. To obtain the specimens for further characterization the material was injected molded in an injection machine Sprinter-11, Erinca S.L. (Barcelona, Spain) (temperature profile of 130, 110, 100 ◦C, from die to hopper). Before and after the processing and characterization, all materials were stored at 25 °C and 50 ± 5% of relative humidity (RH).

#### 2.2.2. Dynamic Mechanical Thermal Analysis

Dynamic Mechanical thermal analysis (DMTA) was conducted in DMA1 Mettler-Toledo (Schwerzenbach, Switzerland) using a single cantilever mode. For the analysis prismatic specimens with rectangular cross-section of 4.5 ± 0.2 × 1 ± 0.2 mm^2^ and a length of 20 ± 2.0 of mm were used. The test was run with an oscillation frequency of 1 Hz at a constant heating rate of 2 °C/min from −100 to 117 °C, with a maximum deformation of 10 µm. The initial static force was 1N. As a result, the dynamic storage modulus (G′) and loss factor (tan δ) curves as a function of temperature are reported. The glass transition temperatures (T_g_) were taken as the maximum values of tan δ curves.

#### 2.2.3. Water Uptake

Water uptake of the TPS-resin blends was determined using flexural test specimens (80 mm × 10 mm × 4 mm) and the parameters specified in ISO 62:2008 standard [38]. Before the test, the samples were dried at 40 °C in an air circulating oven model 2001245 Digiheat-TFT from J.P. Selecta S.A. (Barcelona, Spain). When dried, the samples were weighed (W_0_) and then they were soaked in distilled water. Measurements of water uptake were taken at regular time intervals by removing the samples from the water tank, drying the excess water, and weighing each sample in an analytical balance AG 245 Mettler-Toledo (Barcelona, Spain) with a precision of 0.0001 g. After each measurement, the samples were returned to the water tank. The water absorption (c) was calculated by the difference between the sample weight after an immersion time t (W_t_) and the initial dried sample weight (W_0_) and according to Equation (1) [39].

(1) $$c=\frac{{\;(W}_{t}-W_{0})}{W_{0}}\times 100$$

Three samples of each formulation were evaluated until the saturation weight (W_s_) when no additional weight gain is observed with increasing time. The absorption curves with the mean values are reported.

#### 2.2.4. Surface Characterization, Color, and Wettability

The color parameters of the CIE L*a*b* color space and the yellowness index (YI) were measured using a Colorflex-Diff2 458/08 colorimeter from HunterLab (Reston, VA, USA). Five different points were assessed at aleatory positions over the sample surface. Average values of five YI, L*, a*, and b* coordinates among the standard deviation were reported. Additionally, the total color differences (ΔE) were calculated using TPS as blank [40] and following Equation (2):

(2) $$\Delta E=\sqrt{\Delta a^{2}+\Delta b^{2}+\Delta L^{2}}$$

The surface hydrophobicity of TPS-resin blends was studied through the water contact angle (WCA) of a sessile drop. Analyses were carried out in an optical goniometer EasyDrop-FM140 from Kruss Equipments (Hamburg, Germany) equipped with a camera. A water droplet (≈1.5 µL) was randomly deposited on the surface of the sample with a precision syringe. A capture of the droplet was taken with the camera and transferred to an image software (Drop Shape) for the measurements of the contact angle. Six contact angle measurements were done for each drop. The measurements were conducted at room temperature. The average WCA values are reported among the standard deviation, that in all the samples did not exceed ±3% [41]. 

The significant differences in the surface parameters were statistically assessed at a 95% confidence level according to Tukey’s test using a one-way analysis of variance (ANOVA) employing OriginPro2015 software.

#### 2.2.5. X-ray Diffraction (XDR)

XDR was used to analyze the influence of pine resin derivatives on starch retrogradation. Wide-angle X-ray Diffraction measurements were carried out using a Bruker D8 Advance X-Ray Diffractometer with a linear detector Lynxeye XE. The scattering angles (2θ) covered the ranges from 4° to 50° (θ is the Bragg angle) at a rate of 1°/min. The analyses were done using a 1 mm thick sample with a smooth surface. The XRD patterns of TPS-resin blends are reported in their initial state and after 24 months of storage.

#### 2.2.6. Disintegration under Composting Conditions

The disintegration under composting conditions test was conducted following the parameters of the ISO-20200 standard for a thermophilic degradation period (90 days) [42]. The dry solid residue was prepared by combining 10% commercial compost (Mantillo, Spain), 30% rabbit food, 10% starch, 5% sugar, 1% urea, 4% corn oil, and 40% sawdust. Then, water was added to the mixture to adjust the final water content to 55%. The wet solid residue was placed in plastic containers.

TPS-resin squared films of side 25 cm with an average thickness of 2 mm were prepared for the disintegration study. The TPS-resin blends were compressed molded at 130 °C. Film samples were dried at 40 °C for 48 h before the test. Then, the samples were weighed and placed in wire mesh, which allowed the access of microorganisms and humidity, to facilitate their removal after treatment [43]. The samples were buried 5 cm deep in the wet solid residue in the plastic reactor and incubated under aerobic conditions (58 ± 2 °C) in an oven with circulating air. To guarantee aerobic conditions and relative humidity in the reactor, the compost was mixed gently, and water was added periodically [42,44].

Samples were taken out of the container at different disintegration days (1, 4, 7, 14, 21, 28, 49, 63, 77, and 90). The samples were washed with distilled water, dried in an oven at 40 °C for 48 h, and weighed. A visual evaluation was performed in all the samples when extracted from the composting medium; photographs are presented.

The disintegration degree at different days exposed to the compost medium was calculated by normalizing the sample weight to the initial weight. To determine the time at which 50% of the film is disintegrated (*t*_50_) the disintegrability degree values were fitted using the Boltzmann equation (OriginPro 2015 software) [45,46] following Equation (3):

(3) $$m=\frac{{(m}_{i}-m_{\infty })}{{1+e}^{\left(\frac{1-t_{50}}{d_{t}}\right)}}$$

where m_i_ is the initial mass value measured before the composting test and m_∞_ is the final mass value measured after the final asymptotes of the disintegrability test. d_t_ is a parameter that describes the shape of the curve between the upper and lower asymptotes. The boundary and initial conditions m_i_ is 0% and m_∞_ is 100%. *t*_50_ is known as the half-maximal degradation, and it is the time at which materials disintegrability reaches the average value between m_i_ and m_∞_ [47].

## 3. Results

### 3.1. Dynamic Mechanical Thermal Analysis

**Figure 3:**
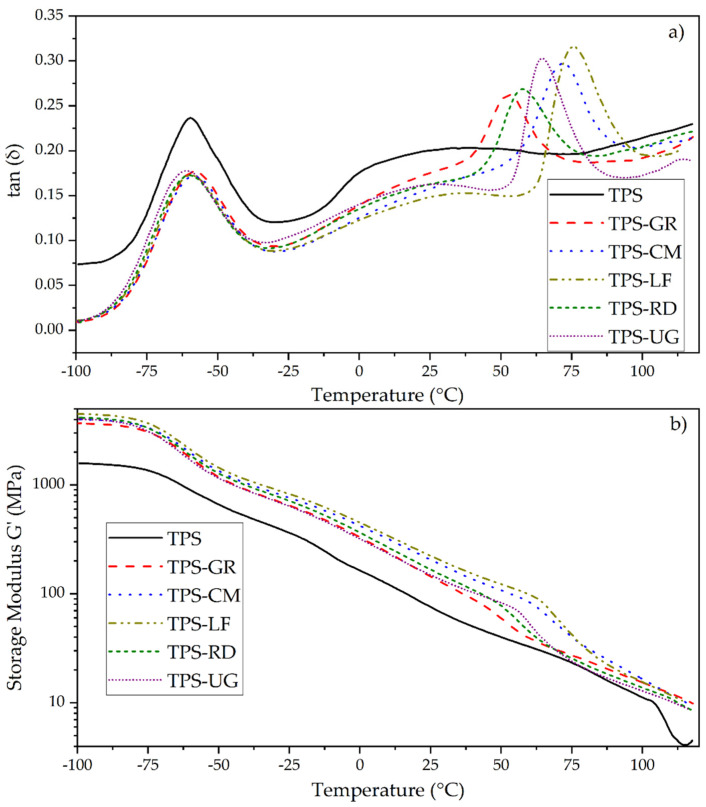
DMTA (**a**) Tan δ; (**b**) Storage modulus.

Figure 3 shows the evolution of the loss factor (tan δ) and storage modulus (G′) with temperature for TPS and its blends with pine resin derivatives. TPS presents a characteristic loss factor curve (black line in Figure 3a) of thermoplastic starch, where a partially miscible system is detected. This is related to phase separation of the starch-glycerol system, resulting in glycerol-rich domains (β relaxation) and starch-rich domains (α relaxation) [48,49]. Two major transitions are seen in the tan δ curves, a narrow and high-intensity peak centered at −60 °C (T_β_), corresponding to the glycerol rich phase, and a broad peak centered at 25 °C (T_α_) corresponding to the starch-rich phase [49,50]. 

It is seen that the addition of pine resin derivatives to TPS did not change the β relaxation transition, as the glycerol content remains constant. However, a decrease in the broadening of the TPS α relaxation transition is observed. The reduction in the broadening suggests that the pine resin derivatives act as solvents to amylose and amylopectin and help to reduce the heterogeneity of the mixture [51]. The effect of this enhanced mixture is noted in the mechanical properties of the materials, reported in previous work [18]. It was determined that the tensile strength is reduced due to the addition of pine resin derivatives. However, Young’s modulus does not change or have a significant increase (TPS-LF) with respect to TPS. The elongation at break increased significantly for TPS-GR and remained invariable with respect to neat TPS for the other formulations [18]. 

In the formulations that contain pine resin and derivatives, the last transition is detected between 50 °C to 100 °C. This transition is observed like a peak at 53 °C in TPS-GR, 58 °C in TPS-RD, 64.6 °C in TPS-UG, 71.6 °C in TPS-CM and 76 °C in TPS-LF in the tan δ curves (Figure 3). These temperatures are consistent with the glass transition temperatures (T_g_) measured in previous work by differential scanning calorimetry (DSC) [18]. The values of this transition detected in DMA and those of T_g_ detected by DSC differ in some degrees because DMA works at higher frequencies than DSC [52]. Additionally, the differences in the temperature values where the peaks are located are due to the chemical structure of each pine resin derivative and it is linked to the degree of mobility that the pine resin additive allows the TPS chains to increase their chain mobility. GR is mainly composed of abietane-type acid while RD has pimarane-type acids in its structure. LF, UG, and CM, are chemically modified rosins with different degrees of modifications. LF is a pentaerythritol ester, UG is a glycerol ester and CM is a maleic anhydride-modified rosin [18]. The shifting of the T_g_ values to higher temperatures suggests a stiffening effect, and thus these materials result in interesting rigid packaging applications intended for hot food [18].

All the mention transitions are reflected with a corresponding decrease in the storage modulus (G′) curves (Figure 3b). At low temperatures (−100 to −70 °C), TPS storage modulus is 1500 MPa and the modulus of all TPS-pine resin derivative formulation is approximately 3900 MPa, a 260% higher than neat TPS. This indicates that in low temperatures, pine resin derivatives increase the elastic component of the matrix [19] by stiffening the structure. Then, the first fall in G′ is observed from −70 °C to −45 °C, which corresponds to glycerol glass transition temperature [52]. From −45 to 40 °C, it is seen that the storage modulus decreases proportionally with the increase of temperature. The behavior of the storage modulus is linked to the gain of mobility of molecular segments in the polymer when the temperature increase. At low temperatures, the molecules have low kinetic energy, therefore the mobility is reduced, and the storage modulus is presented as a plateau. When the temperature increase, so does the kinetic energy and the free volume in the molecular segments, which reduce the elastic component of the material (storage modulus) [53]. In this temperature range (−45 to 40 °C), pine resin derivatives continue to stiffen the TPS structure, increasing the elastic component of the TPS in a constant amount between 200 to 300%. This behavior shows a greater cohesion of the components and reduced heterogeneity, which could be attributed to a chemical interaction between pine resin derivatives and TPS functional groups [19,51]. These chemical interactions are seen in Fourier transform infrared spectroscopy (FTIR) carried out in previous work [18]. 

The second fall in G′ is observed between 45 °C and 75 °C, which shows a loss in the stiffness of TPS pine resin derivatives [54], as the materials reach their glass transition temperature. After this fall, the storage modulus of the TPS-pine resin derivatives formulations equals the storage modulus of neat TPS, which suggests that pine resin derivatives lose their rigidity due to the temperature.

### 3.2. Surface Characterization, Color, and Wettability

Surface color parameters for the CIEL*a*b* space are presented in Table 1. The formulations present significant differences (*p* < 0.5) in all the color parameters respect neat TPS due to the addition of the resin. However, in GR and RD the lightness increased by 55%, in CM and LF it increased 69% and UG produced an increase in the lightness of 80% in TPS.

In all the materials, the a* coordinate (green to red), has negatives values between −1.16 and −1.72 showing the predominance of greenish tones over reddish ones. The b* coordinate (blue to yellow) has positive values between 5.93 and 23.68 which indicates that yellow shades were predominant over the blue ones [40]. All the resins increase the green shade in TPS from 17% to 148%, except for RD that reduces the green shade by 21%. And all the resins increase the yellow coloration of TPS by 100% or more of its initial value. Further, the yellowness index increased in the formulations due to the intrinsic yellowish coloration of the resins, being TPS-CM the formulation with the highest yellowness index and TPS-LF the formulation with the lowest value in this parameter.

The total color difference (ΔE) shows that the addition of any resin caused significant differences in the color of TPS. In all cases, these differences are higher than 2.0, which is the threshold of perceptible color differences for the human eye [10,36]. 

Materials intended for food packaging applications are required to protect foodstuff from humidity. Thus, to obtain information on the hydrophilic/hydrophobic nature of the TPS-resin-based materials, the surface wettability was determined by water contact angle measurements (Table 1). The neat TPS sample showed the lowest water contact angle value due to the number of hydroxyl groups from the native starch as well as glycerol, showing similar values to those reported for corn starch (with 25% amylose content) based formulations plasticized with glycerol [55]. Meanwhile, all TPS-resin blends showed higher WCA values than neat TPS. The surface wettability is dependent on the surface chemistry as well as topographical properties and it seems that the presence of either gum rosin or gum rosin derivatives increases the microstructural roughness (see SEM images in the Supporting Information Figure S1) of the materials. The highest WCA values were observed for TPS-LF and TPS-UG (*p* > 0.05) which were the resins with high molecular weight and high amounts of carbonyl groups. The carbonyl groups are positively interacting with starch hydroxyls groups [18] therefore, there are not hydrophilic groups available to interact with water at the surface of the material. The WCA value was followed by TPS-GR and TPS-RD (*p* > 0.05) which showed lower molecular weight than LF and UG and very similar molecular structures, and finally, the TPS-CM showed the lowest WCA among TPS-resins blend formulations, suggesting that there are some carbonyls and/or carboxylic groups of CM that are interacting with water at the surface. In fact, this formulation showed the less reduction of the -OH group band at 1648 cm^−1^ in the FTIR spectra (see Supporting Information Figure S2) which is related to the bound water, and thus suggest that the carbonyl and/or carboxylic groups of CM have more affinity with water than the rest of resins.

### 3.3. Water Uptake

**Figure 4:**
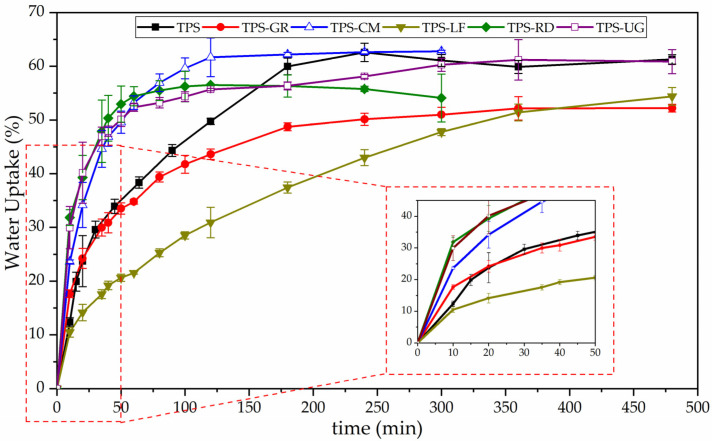
Water uptake of thermoplastic starch (TPS) and formulations with 10 wt.% of gum rosin (GR) and rosin derivatives (CM, LF, RD, UG) with an expanded area in the range from 0 to 480 min.

The evolution of the water uptake of TPS and its formulations with resins are plotted as a function of time in Figure 4. It is observed that all the samples absorbed water. Briefly, the water uptake curves can be divided into two zones. In all the materials, regardless of the used resin, the water uptake is fast in the initial zone (t < 50 min). Whereas, in the second zone, the absorption rate decreases leading to a plateau that corresponds to the saturation [56]. It is observed, the addition of the pine resin derivatives had little effect on the water uptake capacity of TPS. However, the general trend suggests the equilibrium water uptake of TPS decreased with the addition of GR, LF, and RD, while stays in the same value with the addition of UG and CM. This result is related to the number and accessibility of polar groups [57]. In TPS-GR and TPS-RD there are fewer polar groups available to interact with water than in the other formulations, while in TPS-LF the accessibility to the groups is limited due to steric impediments. On the other hand, it is seen that the structure of TPS-CM and TPS-RD did not withstand the expansion and collapsed at 300 min of water uptake, which implies that the interaction inside their structure is weaker than the other formulations, which resisted until 480 min of water uptake before its failure. This could be related to the low toughness and low Young’s modulus of these formulations reported in previous studies [18]. 

### 3.4. X-ray Diffraction Pattern (XDR)

**Figure 5:**
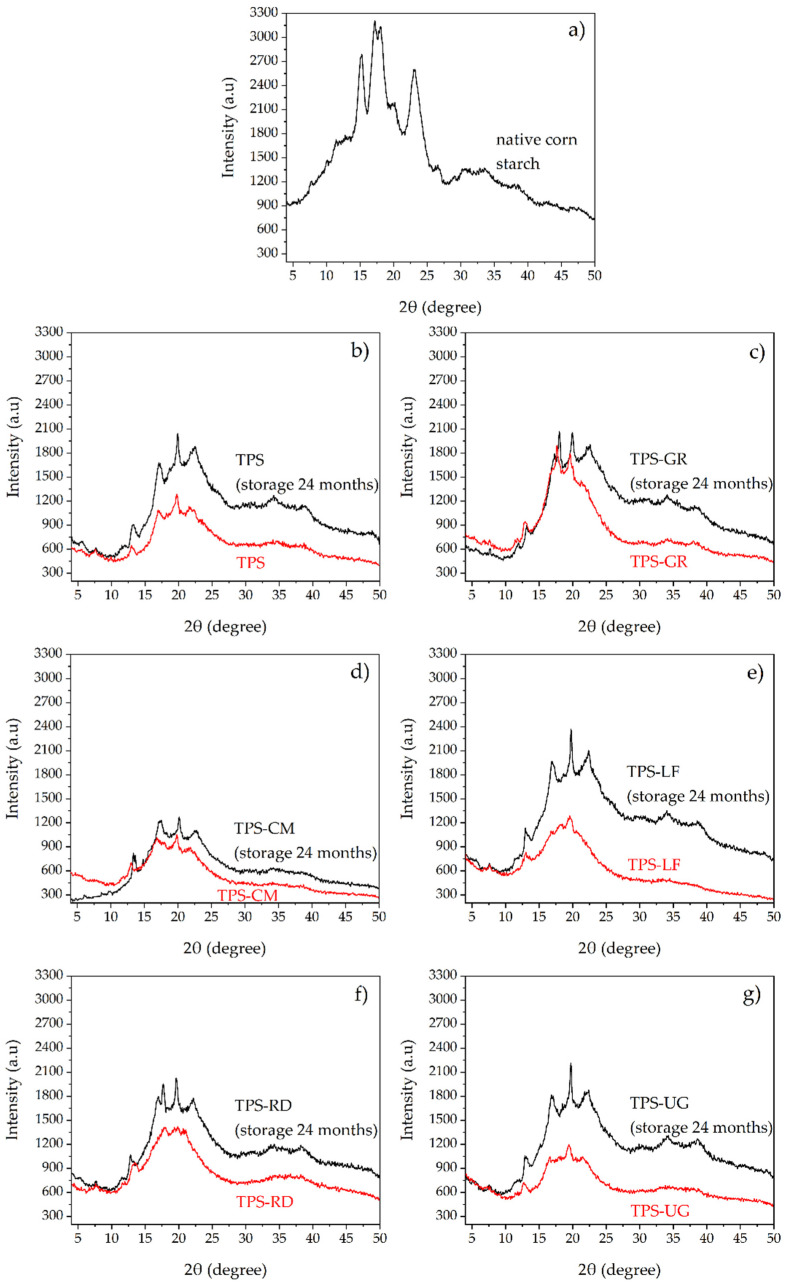
X-ray diffraction intensity scans for (**a**) native corn starch, (**b**) TPS, (**c**) TPS-GR, (**d**) TPS-CM, (**e**) TPS-LF, (**f**) TPS-RD, and (**g**) TPS-UG, in their initial state and after 24 months of storage.

A comparison between the X-ray scattering patterns of native corn starch, TPS, and TPS-pine resin derivatives, before and after being stored for 24 months is presented in Figure 5. Native starch (Figure 5a) show a predominant type A crystallinity, with peaks at 2θ of 15.2°, 17.2°, 18.1°, and 23°, typical of cereals [58]. The scattering pattern of TPS (Figure 5b) presents a broad amorphous baseline, typical of semi-crystalline polymers with a low degree of crystallinity [59]. Moreover, new peaks are observed at 2θ of 13°, 19° and 22°, which is indicative of V_H_ type crystallinity. The broad hump centered on 19° is characteristic of TPS [20]. The V_H_ type crystallinity is assigned to amylose complexed with glycerol formed during thermo-mechanical processing [58]. In the X-ray scattering patterns of TPS-pine resin derivatives before the storage (red curves in Figure 5) it is seen that the peaks corresponding to the A crystallinity (2θ of 15.2°, 17.2°, 18.1°, and 23°) have completely disappeared in TPS-CM, TPS-LF, TPS-RD, and TPS-UG, while residual native corn starch crystallinity is seen in TPS and TPS-GR at 2θ of 17°. This shows that CM, LF, RD, and UT favor the complete disruption of starch granules.

After 24 months of storage (black curves in Figure 5), the formation of B-type crystallinity associated with retrogradation with peaks at 17°, 22°, and 30° is observed in all the formulations [59,60]. The scattering patterns area and intensity of peaks increased in the materials, which shows the crystallization of the structure due to aging. On one hand, LF and UG seem to have a negligible effect on the aging of TPS. On the other hand, the formulations with GR, CM, and RD, have a minimum change in the intensity of X-ray scattering patterns due to storage time. Therefore, it could be said that these resins restrain the aging of TPS due to retrogradation. This could be explained because the molecular structures of GR, CM, and RD are smaller than those of LF and UG (as seen in Figure 1), which allows them to hinder the molecular ordering of TPS. In fact, it has been reported that gums bind water and change the water distribution of the starch-based systems, thus weakening the recrystallization of starch molecular chains [61]. 

### 3.5. Disintegration under Composting Conditions

**Figure 6:**
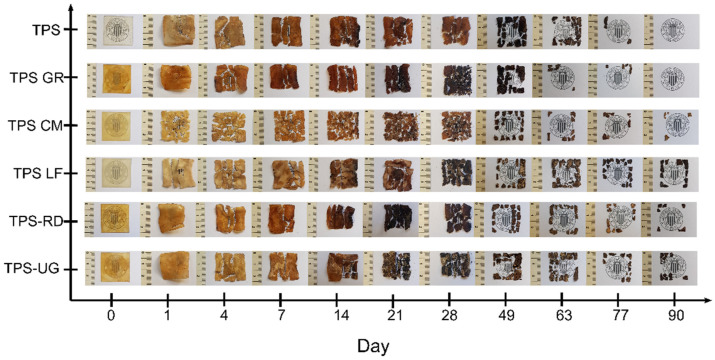
Visual appearance of thermoplastic starch (TPS) and formulations with 10 wt.% of gum rosin (GR) and rosin derivatives (CM, LF, RD, UG) during the disintegration test in controlled compost conditions in terms of the rosin derivatives and the elapsed time.

**Figure 7:**
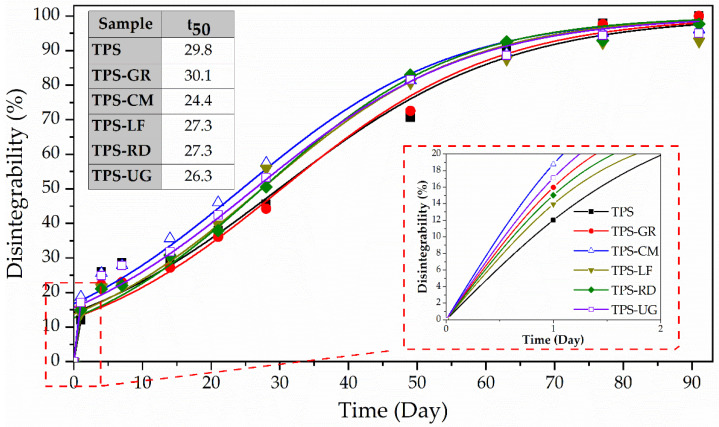
Disintegration degree of thermoplastic starch (TPS) and formulations with 10 wt.% of gum rosin (GR) and rosin derivatives (CM, LF, RD, UG) under controlled compost conditions as a function of time.

Since these materials are intended for sustainable packaging applications, the disintegrability under composting conditions mediated by thermophilic bacteria was assessed to get information regarding the possibility to dispose of these materials with organic wastes. Figure 6 shows the visual appearance of all formulations during the disintegration test, while Figure 7 shows the degree of disintegrability evaluated in terms of mass loss as a function of incubation time by using the Boltzmann equation by the corresponding half-maximal degradation time (see inset Table). The loss of transparency can be observed just after the first day of composting for all TPS-based formulations and these effects were greater after 4 days under composting conditions.

After one day under composting TPS became breakable and lost around 12% of the initial mass. Likewise, all TPS-resin formulations became smaller and breakable, losing between 14% and 19% of the initial mass. The higher mass observed after one day for neat TPS can be related to the fact that starch absorbed water and swelled [55]. The loss of transparency is linked to the changes in the refractive index because of water absorption and the beginning of the formation of low molecular weight compounds as a result of hydrolytic degradation [22,44]. Then, the smaller molecules become susceptible to the enzymatic degradation mediated by microorganisms. TPS changed its color becoming yellowish in 1 day and almost black after 21 days, in good accordance with the literature [22,62]. The presence of pine resin derivatives speeds up the color changes showing a more marked yellow color and reaching the black color between 21 and 49 days depending on the formulation. All formulations showed a similar kinetic pattern of disintegration (Figure 6). TPS-LF and TPS-UG formulation still showed some small pieces of materials after 90 days, while the rest of the formulations virtually disappeared in 77 days. These results are in accordance with the higher hydrophobicity showed by these formulations as it showed the higher WCA. Besides, TPS-LF was the last formulation in being completely disintegrated. The well chemical interaction through hydrogen bonding established in this formulation is also hindering the water diffusion through the bulk, as it was observed in water uptake results (Figure 4), delaying the overall hydrolysis process. Nevertheless, it should be underlined that all the developed TPS-resin formulations were disintegrated in less than 90 days under composting conditions, suggesting their perspective advantage for their industrial production for biodegradable food packaging applications.

## 4. Conclusions

Thermoplastic starch (TPS) was blended with different pine resin derivatives and the obtained materials were characterized focusing on the field of food packaging. It was determined that pine resin derivatives stiffen the structure of thermoplastic starch from −100 °C to 40 °C, which suggests a good cohesion between pine resin derivatives and thermoplastic starch in this temperature range. Besides, the total color of the TPS was significatively influenced due to the addition of pine resin derivatives, which is a consequence of its intrinsic yellowish coloration. Regarding the hydrophobicity, it was determined that pine resin derivatives significatively increase the water contact angle (WCA) of TPS, providing a surface hydrophobicity improvement. WCA values were influenced by the hydrophilic chemical groups available in the structure of the respective pine resin derivative used in the blends. For instance, the blends with pentaerythritol resins (TPS-LF and TPS-UG) present the highest WCA values because the hydroxyl groups have an interaction with the hydroxyl groups in the starch structure, followed by TPS-GR and TPS-RD. Additionally, the water uptake analysis shows that TPS and TPS-pine resin derivatives blends present a high affinity with water, which causes a high water diffusion rate. However, it was seen that GR, LF, and RD decrease the equilibrium water uptake of TPS because these resins present fewer polar groups available. X-ray diffraction analyses showed that CM, LF, RD, and UT favor the complete disruption of starch granules in the fabrication of TPS and that GR, CM, and RD restrain the aging of TPS. The disintegration under composting condition analyses shows that LF and UG delay the disintegration time which could be related to the availability of hydroxyl groups as mentioned in the WCA results. To end, it is worth mention that all the formulations were disintegrated in less than 90 days under composting conditions, which favors their use in biodegradable food packaging applications. Therefore, these materials are interesting for several sustainable rigid food packaging applications, since the increased T_g_ values allow to obtain rigid materials that do not suffer plastic deformation at room temperature, being fully biobased and biodegradable and, thus, aligned with the circular economy approach. Furthermore, the final properties of the material can be focused on a specific area depending on the used pine resin derivative. For applications that seek a film with higher transparency, low water uptake, and high mechanical resistance, the formulation TPS-LF is preferred. However, to restrain the aging due to retrogradation the formulations TPS-CM, TPS-RD, and TPS-GR are ideal. Finally, if it is desired to obtain a disintegration under composting conditions equal to pure TPS, GR can be used in the formulation. 

## Figures and Tables

**Table 1:** Water contact angle and color parameters for the CIEL*a*b* space of neat TPS and the formulations with 10 wt.% GR, CM, LF, RD, or UG.

	WCA	L*	a*	b*	ΔE	YI
TPS	53.0 ± 3.0 ^a^	37.88 ± 0.91 ^a^	−1.47 ± 0.06 ^a^	5.93 ± 0.15 ^a^	0.00 ± 0.00 ^a^	21.04 ± 0.57 ^a^
TPS-GR	80.9 ± 2.9 ^b^	58.11 ± 1.05 ^b^	−1.72 ± 0.08 ^b^	17.55 ± 0.68 ^b^	23.33 ± 1.19 ^b^	44.6 ± 1.17 ^b^
TPS-CM	70.5 ± 1.9 ^c^	64.34 ± 0.70 ^c^	−2.81 ± 0.16 ^c^	23.68 ± 0.80 ^c^	31.90 ± 1.01 ^c^	53.47 ± 1.26 ^c^
TPS-LF	86.4 ± 2.9 ^d^	63.95 ± 0.47 ^c^	−3.64 ± 0.10 ^d^	12.78 ± 0.43 ^d^	27.04 ± 0.54 ^d^	29.35 ± 0.91 ^d^
TPS-RD	77.9 ± 1.7 ^b^	58.89 ± 0.78 ^b^	−1.16 ± 0.18 ^e^	20.85 ± 0.60 ^e^	25.77 ± 0.90 ^d^	51.89 ± 1.13 ^d^
TPS-UG	86.0 ± 0.9 ^d^	68.00 ± 0.94 ^d^	−2.07 ± 0.09 ^f^	17.44 ± 0.49 ^b^	32.26 ± 0.97 ^e^	39.46 ± 0.87 ^e^

^a–f^ Different letters within the same property show statistically significant differences between formulations (*p* < 0.05).

## Data Availability

The data presented in this study are available on request from the corresponding author.

## Supplementary Materials

The following are available online at https://www.mdpi.com/article/10.3390/foods10061171/s1, Figure S1: Scanning electron microscopy (SEM) images of (a) TPS, (b) TPS-GR, (c) TPS-CM, (d) TPS-LF, (e) TPS-RD, and (f) TPS-UG. Figure S2: Fourier transform infrared spectroscopy (FTIR) spectra of: (a) TPS, (b) TPS-GR, (c) TPS-CM, (d) TPS-LF, (e) TPS-RD and (f) TPS-UG.
