# Supplementary material for: Films Based on Thermoplastic Starch Blended with Pine Resin Derivatives for Food Packaging
Source: Foods. 2021 May 23;10(6):1171. doi: 10.3390/foods10061171 (PMC8224774; doi:10.3390/foods10061171)
Supplement: Supplementary file 1 [file foods-10-01171-s001.zip › foods-1219520-supplementary.pdf]

Films based on thermoplastic starch blended with pine resin derivatives for food packaging

Supporting Information

Cristina Pavon [1,*], Miguel Aldas [1,2], Juan López-Martínez[1], Joaquín Hernández- Fernández[3,4], Marina Patricia Arrieta[5,6]

1 Instituto de Tecnología de Materiales (ITM), Universitat Politècnica de València (UPV), 03801 Alcoy, Spain; jlopezm@mcm.upv.es (J.L.-M.)
2 Departamento de Ciencia de Alimentos y Biotecnología, Facultad de Ingeniería Química y Agroindustria, Escuela Politécnica Nacional, 170517 Quito, Ecuador; miguel.aldas@epn.edu.ec (M.A)
3 Research Group in Polymer Science, Engineering and Sustainability, Esenttia, Mamonal Industrial Zona, km. 8, Cartagena, Colombia; hernandez548@hotmail.com (J. H-F)
4 Department of Natural and Exact Sciences, Universidad de la Costa, Calle 58 # 55 – 66, Barranquilla, Colombia
5 Departamento de Ingeniería Química Industrial y del Medio Ambiente, Escuela Técnica Superior de Ingenieros Industriales, Universidad Politécnica de Madrid (ETSII-UPM), Calle José Gutiérrez Abascal 2, 28006, Spain; m.arrieta@upm.es
6 Grupo de Investigación: Polímeros, Caracterización y Aplicaciones (POLCA), 28006 Madrid, Spain.
* Correspondence: crisppavonv@gmail.com (C.P.); Tel.: +34-644343735 (C.P.)

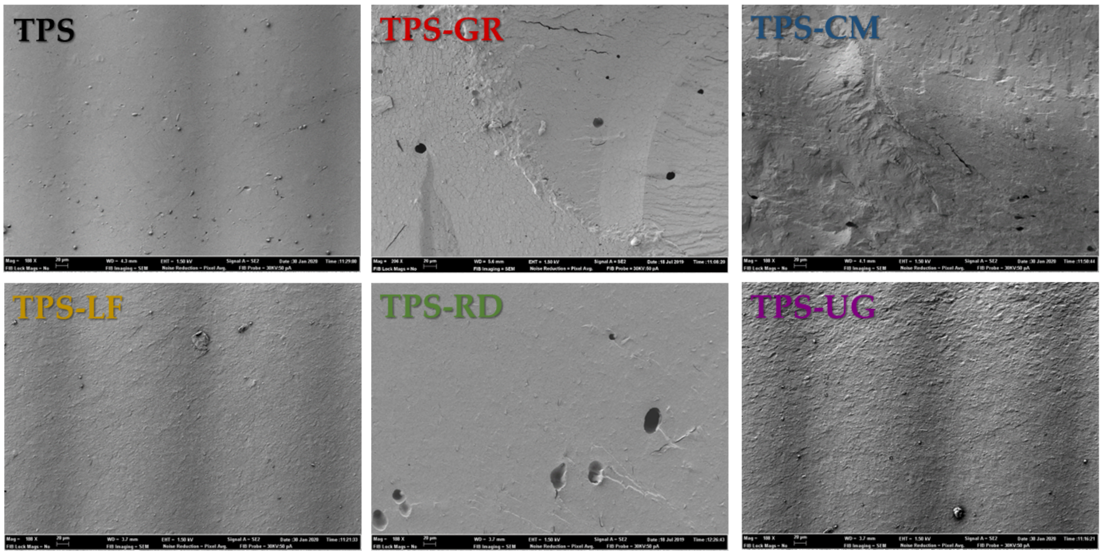

Figure S1. Scanning electron microscopy (SEM) images of: (**a**) TPS, (**b**) TPS-GR, (**c**) TPS-CM, (**d**) TPS-LF, (**e**) TPS-RD and (**f**) TPS-UG.

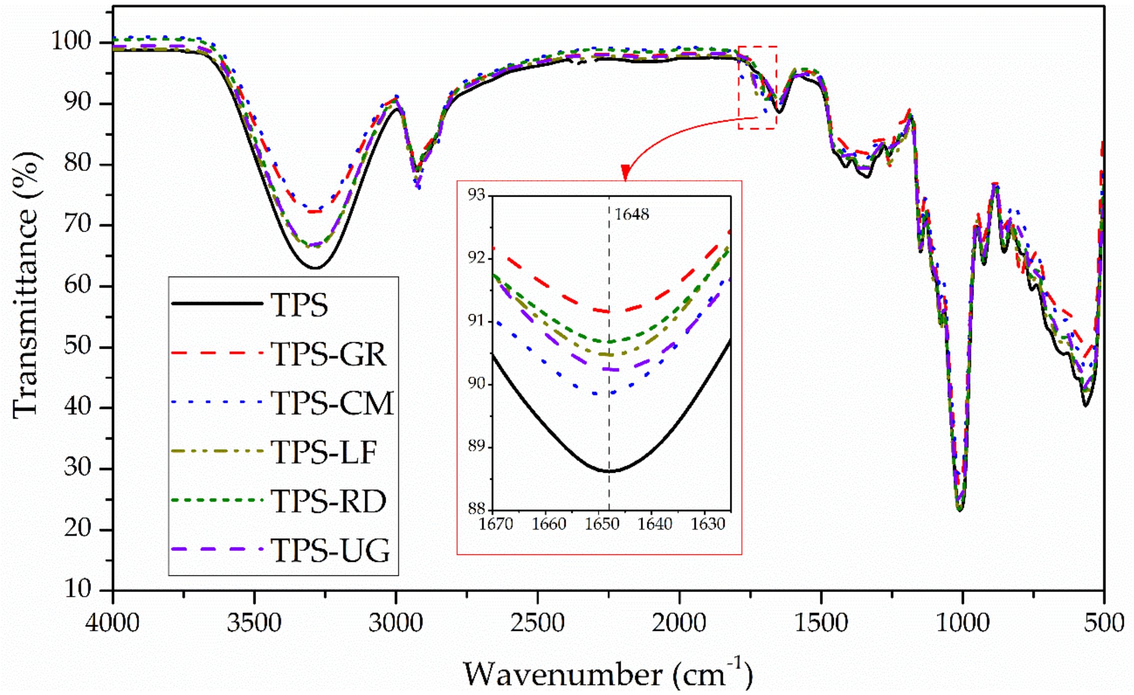

Figure S2. Fourier transform infrared spectroscopy (FTIR) spectra of: (**a**) TPS, (**b**) TPS-GR, (**c**) TPS-CM, (**d**) TPS-LF, (**e**) TPS-RD and (**f**) TPS-UG.
